# Generating Civically-Engaged Undergraduate Student Scientists in General Education Classrooms

**DOI:** 10.3389/fpsyg.2019.00664

**Published:** 2019-04-02

**Authors:** Tara T. Lineweaver, Tonya R. Bergeson

**Affiliations:** ^1^Department of Psychology, Butler University, Indianapolis, IN, United States; ^2^Department of Communication Sciences and Disorders, Butler University, Indianapolis, IN, United States

**Keywords:** civic engagement, general education, undergraduate research, student scientists, teaching-led research

Historically, teaching and research have been closely intertwined in academic settings (e.g., Brown and McCartney, 1998; Deakin, 2006; Harland, 2016; Jucks and Hillbrink, 2017). Because the pressure to be both an excellent lecturer and a renowned researcher can lead faculty to perceive teaching or research as a burden, several approaches to linking teaching and scholarship have been empirically investigated (e.g., Freestone and Wood, 2006; Dexter and Seden, 2012; Pan et al., 2014; Harland, 2016; Jucks and Hillbrink, 2017). This “teaching-research nexus” (Neumann, 1994) connection can assume many forms (Brew, 2010).

Most research in this field has focused on how faculty can introduce students to their own scholarship as it relates to the content of their courses (Moses, 1990; Brew, 1999; Dexter and Seden, 2012). Two past studies found that students value faculty enthusiasm when they are teaching their area of expertise, appreciate learning from well-known researchers, and recognize the enhanced credibility of faculty and institutions with strong scholarship records (Jenkins et al., 1998; Healey et al., 2010). At the same time, students may perceive disadvantages; they may believe that faculty who are strong scholars are less available and accessible, prioritize research over teaching, and do not afford students ownership of the research conducted at their institution.

Involving students directly in faculty scholarship is an alternative approach that could alleviate these issues. This approach builds valuable mentoring relationships between faculty and student collaborators (Eby et al., 2008), offers students “the excitement and enthusiasm of inquiry,” teaches research skills (Dexter and Seden, 2012), and gives students a better understanding of the scientific process (Pan et al., 2014). Although these advantages reinforce the value of involving students as collaborators on faculty-led scholarship, faculty are often limited in the number of students they can mentor due to other demands on their time (Healey et al., 2010).

A third, potentially equally impactful but likely farther-reaching way to link teaching and research is designing classes that develop student scientists by involving them in faculty scholarship within the context of their courses. Harland (2016) investigated this “teaching-led research” approach within an Ecology curriculum designed to replace the previous method of teaching majors content early in their undergraduate years and introducing inquiry-based learning later. He found that teaching students to be researchers in the classroom was beneficial. Beyond the advantages of involving undergraduates in research more generally, faculty became more excited and expanded their thinking about their scholarship, in part due to the unique perspectives offered by their students.

Designing classes that directly support faculty research also has potential pitfalls. Few universities have reward systems in place for this type of approach, and these types of courses may lead students to believe that faculty are promoting their own interests rather than taking the students' educational needs into account (Harland, 2016). Likewise, Pan et al. (2014) warn against an overcrowding of the curriculum; focusing on content as well as on teaching students how to implement the research process consumes vast amounts of class time. Thus, these types of efforts may fit best in courses where mastering content is not the primary objective, but rather introducing students to a discipline or to scientific inquiry more generally is the goal.

To capitalize on the benefits and overcome the limitations of teaching-led research, we adopted this approach in a general education course. Unlike the previous examples (Dexter and Seden, 2012; Pan et al., 2014; Harland, 2016), we taught students from diverse backgrounds about science early in their academic career—a core curriculum class designed for first- and second-year non-science majors—and trained them to serve as student scientists in the community. This approach not only developed students' scientific knowledge and skills and made them stakeholders in the research process, but also allowed us to progress on our own scholarship without detracting from our teaching (Dexter and Seden, 2012; Harland, 2016). Here, we outline eight steps to establish a teaching-led research course based on our experiences and offer ideas for expanding the model beyond one semester to promote publishable student research.

## Design a Research Project

For a successful balance of teaching and research, we suggest designing a research project that allows faculty to train students as student scientists quickly and that demonstrates the value of science to non-science majors. A project that links science to the community helps students see the applied value of the scientific approach. Our “Music First!” project combines music, psychology, pharmacy, and communication sciences and disorders toward addressing a key community issue: older adult nursing home residents nationwide experience dementia-related symptoms that lead physicians to prescribe medications that can worsen cognitive decline and cause physical harm (Reus et al., 2016). We investigate whether playing individualized music playlists for these residents improves their quality of life, decreases their dementia-related symptoms, and leads to a reduction in their medications.

## Design a Teaching-led Research Course

To reach a diverse student population, we created a class that fit into the natural science portion of our core curriculum. This five credit-hour co-taught course included three hours of lecture each week plus a three-hour lab. The course focused around the science of music, the auditory system, emotion, memory, and dementia. While covering this content, we also taught students the scientific method and trained them to act as student scientists with responsibility for conducting the “Music First!” research project.

## Introduce Students to the Scientific Method

We taught students the scientific method by engaging them as student scientists from the beginning of the semester to the end. Early in the semester, students learned about the scientific method, research design, data collection, reliability and validity of data, and research ethics, which prepared them to understand our specific research project.

## Introduce Students to the Research Project

During the lab portion of the course, we introduced students to the research problem and familiarized them with the research methods we designed to address the issue. We trained them in the necessary data collection techniques and in how to interact effectively and comfortably with the older adult dementia patients they would serve. To accomplish this training quickly, we created online modules for students to complete outside of the classroom that prepared them for class-based training sessions.

By Week 5 of the 16-week semester, students spent their lab time each week visiting the nursing home, playing personalized music playlists for the residents, and collecting data for the project. Specifically, they evaluated nursing home residents' sundowning symptoms prior to and after music listening.

## Generate Hypotheses

During Week 7, students generated their own hypotheses related to the study. We asked individual students to brainstorm multiple hypotheses, submitted all of these hypotheses to a class vote, and assigned groups of 4–5 students to focus on the five hypotheses that were of greatest interest to the class.

## Review the Literature

We trained students to search the literature, read scientific articles, and understand the key components of a published manuscript. They completed an assignment in which each student located one article related to their groups' hypothesis and described the goals, hypotheses, key variables, research design, results, conclusions, limitations and future directions of the study.

## Analyze the Data

During Week 13, we provided each group of students a dataset containing the variables that pertained to their sample and hypothesis. We introduced students to data analysis and statistics. Working in groups, they investigated their assigned hypotheses by analyzing the data they had collected.

## Prepare Presentations

During the last few weeks of the course, students prepared an end-of-the-semester oral presentation summarizing relevant background research built from their literature review assignment, their research goals and hypotheses, their approach to analysis, and their results, conclusions and the implications of their findings. The students presented their talks in class, with the faculty and community members involved in the research in attendance. Although our students did not present their work at local undergraduate or national conferences, their presentations certainly were of a caliber to do so, and this model could easily expand to include external presentations or publishable papers as culminating events.

**Figure 1:**
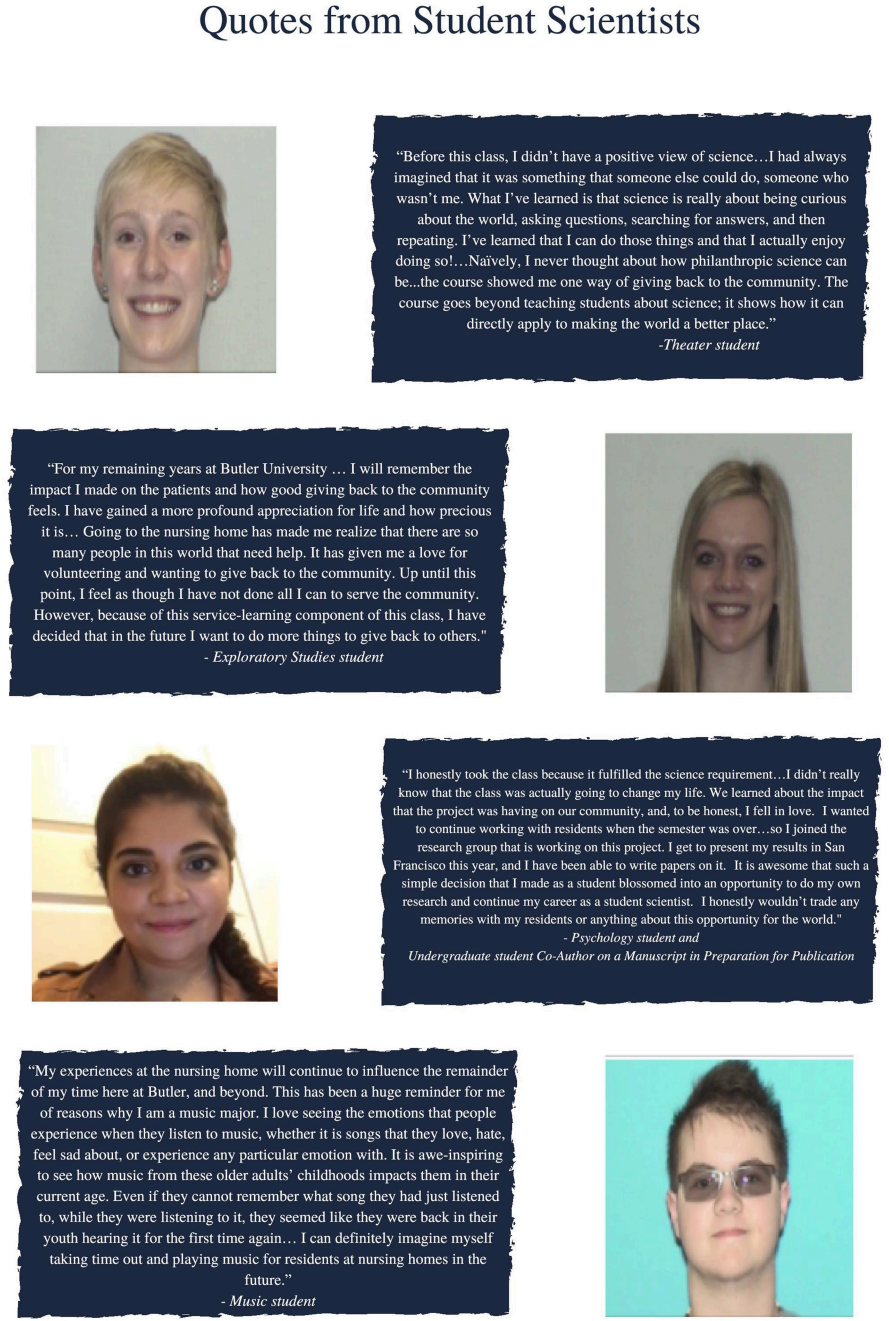
Quotes from student scientists' post-course reflections that demonstrate their enhanced appreciation of science, civic engagement, and their majors as a result of the course. Written informed consent was obtained from the individuals pictured in this figure for the publication of these images.

At the end of the semester, our students reflected on the course and their role as student scientists. Consistently, students valued the teaching-led research approach and appreciated the opportunity the course gave them to both serve their community and grow as scientists, regardless of their discipline. Figure 1 includes quotes from students and demonstrates the value of this model toward generating civically-engaged undergraduate student scientists in general education classrooms.

### Publishable Research

Although in one semester with non-science majors, we did not accomplish professional presentations or publications, we envision two paths for expanding this model to achieve that ambitious goal. The first approach, which we have not yet tried, has great potential to involve a large number of students in publishable research. The second approach, we have used with less far-reaching, but no less successful, outcomes.

A first path to student publications would be to add a required, optional, or by-invitation-only second semester course focused on further developing the skills the students established in their first semester (e.g., expanding their search and review of the literature) and on teaching them additional skills necessary for generating a manuscript (e.g., the basics of scientific writing). Together, this would allow them to translate their presentations into manuscript form. Those who enroll could focus their manuscripts on the strongest hypotheses and results from the first-semester projects or could continue the work they, specifically, started in the first semester. Because data collection would be complete and the results previously analyzed, we believe that taking a step-by-step approach with 4 weeks spent on each section of the paper would allow a full manuscript to be written within the confines of a semester.

The second path to moving students from the course to published co-authors is to recruit or accept selected students into research labs at the conclusion of the course. Across the approximately 100 students we taught during four semesters, 10 chose to continue collaborating with us. Some students approached us asking how to continue their work with nursing home residents; we actively recruited other highly engaged students to join our labs. While some have simply continued to collect data, others have served as project leaders, making this or related studies the primary focus of their ongoing undergraduate research. To date, these 10 students have authored three presentations at undergraduate conferences and four at state/national conferences. One is co-authoring a manuscript that is currently in preparation.

Based on our experiences, we are confident that this teaching-led research approach offers great promise as a means to link scholarship and teaching because it addresses several pressing issues in settings where faculty must both teach well and be productive researchers (Pan et al., 2014). By engaging undergraduate students in publishable research, students become collaborators in the classroom rather than passive learners of information (Ramsden, 2009). This challenges students to think (Harland, 2016) and actively engages them in their own learning (Pan et al., 2014). Additionally, teaching-led research helps to address the switch from valuing teaching to valuing research that is occurring at many midsize institutions, where growing student enrollments demand more time dedicated to teaching and leave only limited time for highly prized scholarship (Dexter and Seden, 2012; Harland, 2016). Thus, teaching-led research may be particularly useful at institutions with high teaching loads because it directly links teaching to productive, publishable scholarship. At the same time, this approach may fit well at larger institutions where undergraduate students otherwise may face limited opportunities to engage in productive scholarship due to a shortage of available spots for research lab experiences. Regardless of the setting, by designing courses in a way that involves students in scholarship within their classes, a broader cohort of students can grow in their research expertise. Expanding student involvement beyond one semester could also result in many undergraduate students co-authoring professional presentations and manuscripts. Perhaps most importantly, this model gets students off campus and into the community, conducting applied research with real-world applications that are highly recognizable while promoting publishable faculty and student scholarship.

## Author Contributions

All authors listed have made a substantial, direct and intellectual contribution to the work and have approved it for publication.

### Conflict of Interest Statement

The authors declare that the research was conducted in the absence of any commercial or financial relationships that could be construed as a potential conflict of interest.
